# Resveratrol inhibits TGF-β1–induced fibrotic effects in human pterygium fibroblasts

**DOI:** 10.1265/ehpm.23-00020

**Published:** 2023-10-21

**Authors:** Jianwu Fan, Shuang Wei, Xiaoyan Zhang, Li Chen, Xin Zhang, Yaping Jiang, Minjie Sheng, Yihui Chen

**Affiliations:** 1 Department of Ophthalmology, Yangpu Hospital, School of Medicine, Tongji University, Shanghai 200090, China; 2 Department of Ophthalmology, Huashan Hospital, Fudan University, Shanghai 200040, China; 3 Center for Clinical Research and Translational Medicine, Yangpu Hospital, School of Medicine, Tongji University, Shanghai 200090, China; 4 Department of Ophthalmology, Yangzhi Rehabilitation Hospital, School of Medicine, Tongji University, Shanghai 201600, China

**Keywords:** Resveratrol, Pterygium, Fibrosis, TGF-β1, Smad3, AKT, p38 MAPK

## Abstract

**Background:**

Resveratrol is a polyphenolic phytoalexin which has the properties of anti-oxidant, anti-inflammatory and anti-fibrotic effects. The aim of this study was to investigate the anti-fibrotic effects of resveratrol in primary human pterygium fibroblasts (HPFs) and elucidate the underlying mechanisms.

**Method:**

Profibrotic activation was induced by transforming growth factor-beta1 (TGF-β1). The expression of profibrotic markers, including type 1 collagen (COL1), α-smooth muscle actin (α-SMA), and fibronectin, were detected by western blot and quantitative real-time-PCR after treatment with various concentrations of resveratrol in HPFs to investigate the anti-fibrotic effects. Relative signaling pathways downstream of TGF-β1 were detected by Western blot to assess the underlying mechanism. Cell viability and apoptosis were assessed using CCK-8 assay and flow cytometry to evaluate proliferation and drug-induced cytotoxicity. Cell migration and contractile phenotype were detected through wound healing assay and collagen gel contraction assay.

**Results:**

The expression of α-SMA, FN and COL1 induced by TGF-β1 were suppressed by treatment with resveratrol in dose-dependent manner. The Smad3, mitogen-activated protein kinase (p38 MAPK) and phosphatidylinositol-3-kinase (PI3K) / protein kinase B (AKT) pathways were activated by TGF-β1, while resveratrol attenuated those pathways. Resveratrol also inhibited cellular proliferation, migration and contractile phenotype, and induced apoptosis in HPFs.

**Conclusions:**

Resveratrol inhibit TGF-β1-induced myofibroblast activation and extra cellular matrix synthesis in HPFs, at least partly, by regulating the TGF-β/Smad3, p38 MAPK and PI3K/AKT pathways.

## Introduction

Pterygium is a fibrovascular degenerations of the conjunctiva that extends into the corneal limbus [1]. It can cause significant damage to visual function, triggering irritation [2], inflammation [3] and astigmatism [4]. Currently, there is no effective drug or conservative treatment for advanced pterygium, and surgery remains the best treatment option. However, simple excisions result in a high recurrence rate (38–88%) [5]. Recurrent pterygium with excessive fibrovascular tissues may increase surgical difficulty, risk, and cause symblepharon and limited eye movement. Therefore, it is crucial to develop more targeted medical treatments to prevent pterygium recurrence after surgery.

The expression of α-SMA is up-regulated in cultured human pterygium fibroblasts (HPFs) derived from severe pterygium compared with mild pterygia, indicating the accumulation of myofibroblasts in pterygium development [6]. Numerous studies have demonstrated the overexpression of transforming growth factor-β1 (TGF-β1) in pterygium tissues compared to normal conjunctival tissues [7, 8]. In this study, TGF-β1 was used as an inducer to stimulate the fibrosis of HPFs.

Resveratrol (3, 5, 4′-trihydroxy-trans-stilbene, Res) is a polyphenolic phytoalexin, with several biological activities, including inhibiting platelet aggregation [9], anti-oxidant [10], anti-inflammatory [11], anti-apoptotic [12] and anti-tumor properties [13]. Moreover, evidence suggests that resveratrol protects against numerous fibrotic diseases, including liver fibrosis [14], cardiac fibrosis [15], and pulmonary fibrosis [16].

In the present study, we aimed to investigated the antifibrotic effects of resveratrol on TGF-β1-induced HPFs and explore the underlying mechanisms involved in the observed effects.

## Materials and methods

### Patients and primary HPFs cultures

The pterygium tissues were obtained from 8 patients (three males and five females) with average age of 57.88 ± 11.97 years (range, 34–71 years) during surgical excision of pterygium combined with limbal-conjunctival autograft at our hospital. All tissue samples were obtained after approval from the institutional review board of Yangpu Hospital. This research followed the tenets of the Declaration of Helsinki. All patients participated in this research provided written informed consent before surgery after being informed of possible consequences.

The excised pterygium head samples were placed in culture medium (Dulbecco’s modified Eagles’ medium nutrient mixture [DMEM/F12, 1:1]; Gibco Life Technologies, NY, USA). The samples were immersed in 10% penicillin/streptomycin (Gibco Life Technologies) for 5 minutes and washed three times with PBS (Hyclone Laboratories, Utah, USA), cut into small pieces, and then placed in 60-mm culture dishes with DMEM/F12 containing 20% fetal bovine serum (FBS; Sciencell Research Laboratories, CA, USA). The cells were maintained at 37 °C in 5% CO_2_. After migration from the explant, the cells were dissociated using 0.25% trypsin with EDTA (Gibco Life Technologies) and propagated at a 1:2 ratio. Cells between the third and seventh passages were used for subsequent experiments. All the experiments were performed at least three times.

### Drug treatments of cells

The HPFs were seeded into culture plates in DMEM/F-12 with 10% FBS. The cells were starved for 8 hours after attachment. For the antifibrotic effects of resveratrol studies, the resveratrol (purity > 98%; Sangon Biotech Co. Ltd. Shanghai, China) was dissolved in DMSO, and dispensed into a 10 Mmol stock solution stored at −20 °C. It was then added into cell medium with four final concentrations (25, 50, 75 and 100 µM) when used to treat cells for 48 hours with 10 ng/mL TGF-β1 (PeproTech, NJ, USA). For inhibitor studies, the inhibitors SB203580 (20 µM, p38 MAPK inhibitor), LY294002 (30 µM, PI3K/AKT inhibitor) and SIS3 HCI (20 µM, Smad3 inhibitor) which all purchased from Selleck were added with TGF-β1 after the starvation period. TGF-β1 was dissolved in RNase-free water, the inhibitors were dissolved in DMSO. The same volume of solvents (RNase-free water, or DMSO) was used as controls or vehicle.

### Western blot assay

Following treatment, cell lysates were extracted from the cells through lysis in ice-cold Ripa buffer (Yeasen, Shanghai, China). The protein concentration of lysates was determined by a bicinchoninic acid (BCA) protein assay kit (Thermo Fisher Scientific). Equal amounts (20–30 µL) of total proteins were separated by sodium dodecyl sulfate polyacrylamide gel electrophoresis (SDS-PAGE) and transferred to polyvinylidene fluoride membrane (PVDF, Millipore, Merck KGaA, Darmstadt, Germany). Membranes with protein samples were incubated with 5% skimmed milk dissolved in Tris-buffered saline with 0.1% Tween-20 (TBST) for 1 h and subsequently incubated overnight with primary antibodies at 4 °C. The primary antibodies used in the experiment were as follows: α-SMA (1:1000, Cell Signaling Technologies [CST], Danvers, MA, USA), COL1 (1:1000, CST), Fibronectin (1:200, Santa Cruz, CA, USA), GAPDH (1:2000, Abcam), Smad4 (1:1000, CST), p-Smad3 (1:800, CST), Smad3 (1:800, CST), p-AKT (1:1000, Proteintech Group, WUHAN SANYING, Wuhan, China), AKT (1:1000, Proteintech Group), p-ERK1/2 (1:1000, CST), ERK1/2 (1:1500, CST), p-p38 MAPK (1:1000, CST) and p38 MAPK (1:1000, Proteintech Group). The membranes were washed with TBST and incubated with species-specific IgG labelled with horseradish peroxidase-conjugated secondary antibodies (1:5000, Cell Signaling Technology) for 1 h. Finally, immunoblotting images were captured by the ChemiDoc imaging system (Bio-RAD, Hercules, CA, USA) using enhanced chemiluminescence (ECL) (Millipore). Densitometry quantification of the bands were quantified by ImageJ software (National Institutes of Health, Bethesda, MD, USA). Relative protein levels were calculated by referencing to the amount of GAPDH protein.

### Quantitative real-time polymerase chain reaction

The cells were harvested after treatment for 48 hours and total RNA was extracted using the TRIzol reagent (Takara, Kusatsu, Japan). Next, the RNA was reverse-transcribed into complementary DNA using the 1st Strand cDNA synthesis kit (Yeasen, Shanghai, China) according to the instructions provided by the manufacturer. RT-PCR was performed using the StepOnePlus real-time PCR detection system (Applied Biosystems Life Technologies, Thermo Fisher Scientific). The reaction contained SYBR Green qPCR mix (high ROX) (Yeasen), cDNA, primers, and nuclease-free water (total reaction volume: 20 µl). False positive signals caused by primer dimers were excluded by dissociation curve analysis. All mRNA levels were normalized to those of GAPDH, and the calculations were performed using the 2-ΔΔCT method. The data are presented as fold-changes. The primers for PCR analysis were as follows: GAPDH, forward 5′-AAGAAGGTGGTGAAGCAGGC-3′ and reverse 5′-TCCACCACCCTGTTGCTGTA-3′; α-SMA, forward 5′-GGCATTCACGAGACCACCTAC-3′ and reverse 5′-CGACATGACGTTGTTGGCATAC-3′; FN forward 5′-CGGTGGCTGTCAGTCAAAG-3′ and reverse 5′-AAACCTCGGCTTCCTCCATAA-3′; COL1 forward 5′-GAGGGCCAAGACGAAGACATC-3′ and reverse 5′-CAGATCACGTCATCGCACAAC-3′.

### Cell viability test

The HPFs were resuspended and seeded into 96-well plates at a density of 5000 cells/100 µL per well and incubated at 37 °C. After cell attached, the culture medium was replaced with the indicated reagent. The cell viability was measured by the CCK-8 assay (Yeasen) according to the kit manufacturer’s protocol following treatment for 48 hours. The cells were incubated for 2 h with 10 µl CCK-8 reagent in each well. Finally, assessment of cell viability was done by measuring the optical density (OD) at 450 nm using a microplate reader (Thermo Fisher Scientific).

### Apoptosis test

The apoptosis of HPFs was assessed using flow cytometry. After treatment with TGF-β1 or plus various concentrations (50, 100 µM) of resveratrol for 48 hours, the HPFs were washed with PBS twice and resuspended in binding buffer at a concentration of 1 × 10^6^ cells/mL. Subsequently, they were stained with fluorescein isothiocyanate (FITC)-conjugated annexin-V using a Annexin V-FITC/PI Apoptosis Detection Kit (Yeasen) at room temperature for 15 min. Data were acquired using a BD Accuri C6 system (BD Biosciences, San Jose, CA, USA) and analyzed with the BD Accuri C6 software (BD Biosciences).

### Wound healing assay

The HPFs were seeded into culture plates and incubated for 24 h to reach 90% confluency in each well. Then, scratch wounds were induced using a sterile pipette tip (200 µl), and the plates were washed with PBS three times to remove loose or dead cells. The HPFs were subsequently treated with TGF-β1 only or plus with different concentrations of resveratrol in 1% serum medium. At the indicated time points, the wound gaps in the same location were photographed using phase-contrast microscopy (Olympus, Tokyo, Japan). The ImageJ software was used to determine the wound area by three masked colleagues, and the data were quantified by comparing with the wound areas observed at 0 h.

### Collagen gel contraction assay

The HPFs-embedded collagen gel contraction assay was performed as a previously described method [17, 18]. Briefly, HPFs were resuspended in serum-free DMEM medium on ice, then mixed with neutralized collagen solution (type I collagen from rat tail tendon; Corning Inc., Corning, NY, USA) at the finally concentration of 3 × 10^5^ cells/mL. Cell-collagen solution were transferred into 24-well plates coated with 1% FBS overnight. The collagen gels were detached from the bottom of the culture plate after incubated at 37 °C for 45 minutes. Culture medium with TGF-β1 only or different concentrations (50, 100 µM) of RSV was added and incubated at 37 °C. Subsequently, the surface area of each collagen gel was observed and recorded, and the percentage of gel contraction was calculated by measuring the size of gel compared with the surface area of wells using the ImageJ software. The experiment was repeated for three times and the last results was selected.

### Statistical analysis

All data are presented as the mean ± standard error of the mean. Statistical significance across groups was determined by one-way analysis of variance (ANOVA) or Student’s t-test with the SPSS statistical software (version 18.0; IBM Corporation, Armonk, NY, USA). Differences between groups were considered significant when P < 0.05.

## Results

### Resveratrol significantly reduced TGF-β1-induced synthesis of ECM proteins and myofibroblast differentiation in HPFs

**Fig. 1:**
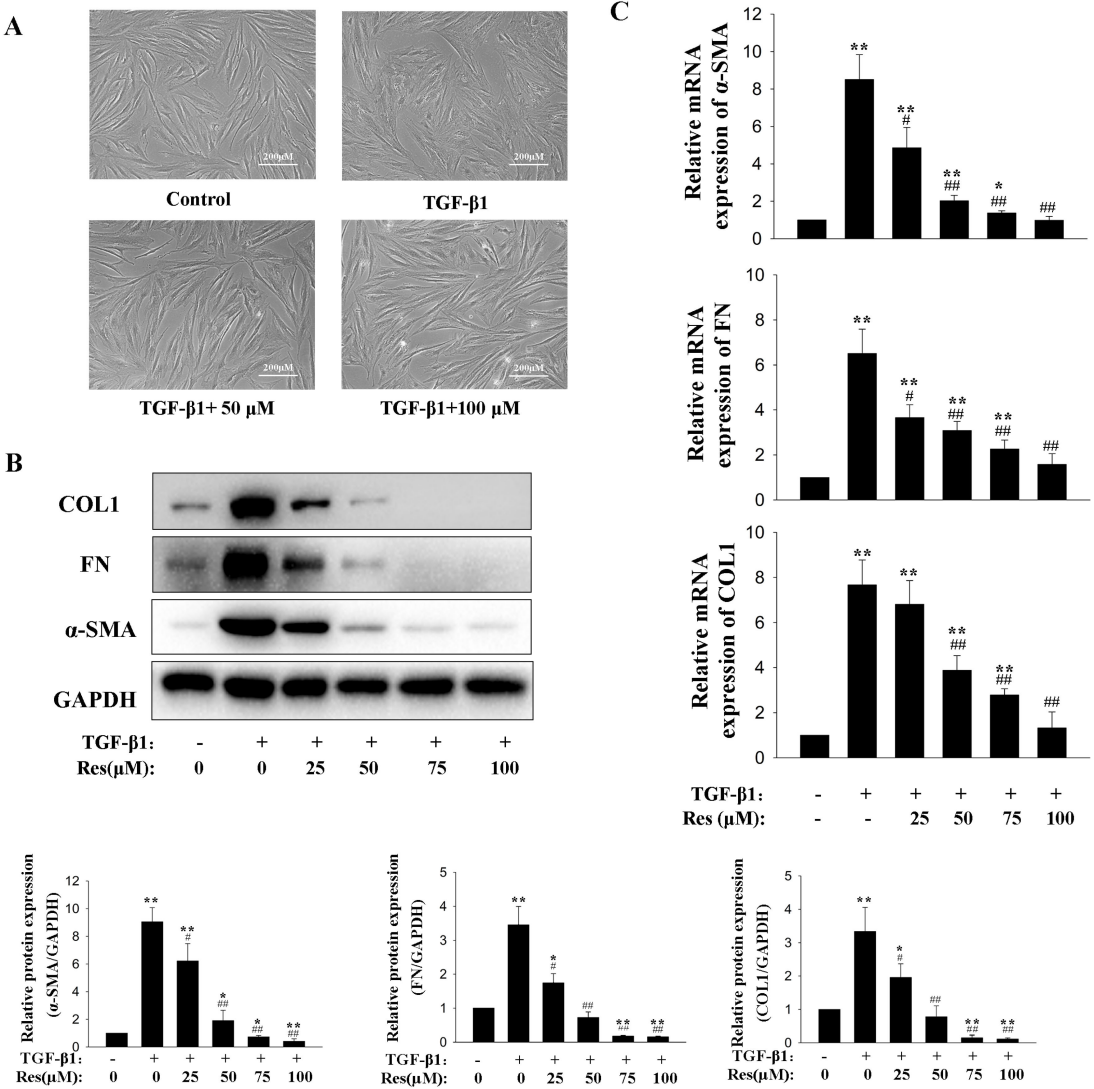
Effects of resveratrol on profibrotic activation, expression of myofibroblastic markers and ECM proteins in HPFs. HPFs were treated with vehicle, TGF-β1 (10 ng/mL) only or plus different concentration of resveratrol for 48 hours. (A) Phase-contrast micrographs showing the morphology change in HPFs. (B) Representative Western blot images of α-SMA, FN and COL1 protein expression. Quantitative analysis of immunoblots with summary data from three independent experiments. Total protein levels were normalized to those of GAPDH (loading control). (C) Quantitative analysis of mRNA expression of α-SMA, FN and COL1. Error bars represent SEM. *P < 0.05, **P < 0.01, versus control; ^#^P < 0.05, ^##^P < 0.01 versus with TGF-β1, n = 3. Abbreviations: α-SMA, α-smooth muscle actin; COL1, type 1 collagen; FN, fibronectin; GAPDH, glyceraldehyde-3-phosphate dehydrogenase; HPF, human pterygium fibroblast; qRT-PCR, quantitative real-time polymerase chain reaction; SEM, standard error of the mean; Res, resveratrol; TGF-β1, transforming growth factor-β1.

The morphology and fibrosis-relative gene expression of HPFs were observed and tested after treated with TGF-β1 (10 ng/mL) or plus different concentration of resveratrol (25, 50, 75, 100 µM) for 48 hours. The original HPFs showed the elongated shuttle or dendritic shape of fibroblasts. After incubation with TGF-β1, the morphology of HPFs were change into spindle shaped accompanied with abundant of filamentous structure. Treatment with resveratrol attenuated the morphology change induced by TGF-β1 (Fig. 1A). Then, the expression of α-SMA, and fibronectin (FN), type I collagen (COL1), the ECM proteins were detected by Western blot. There was a marked increase in the expression α-SMA, FN and COL1 relative to vehicle-treated control group after treatment with TGF-β1. Resveratrol treatment for 48 hours dramatic decreased the expression of α-SMA, FN and COL1 stimulated by TGF-β1 (Fig. 1B). The expression of α-SMA, FN and COL1 in mRNA level were also confirmed by q-PCR (Fig. 1C).

### Effects of resveratrol on the signaling pathways in TGF-β1-induced HPFs

**Fig. 2:**
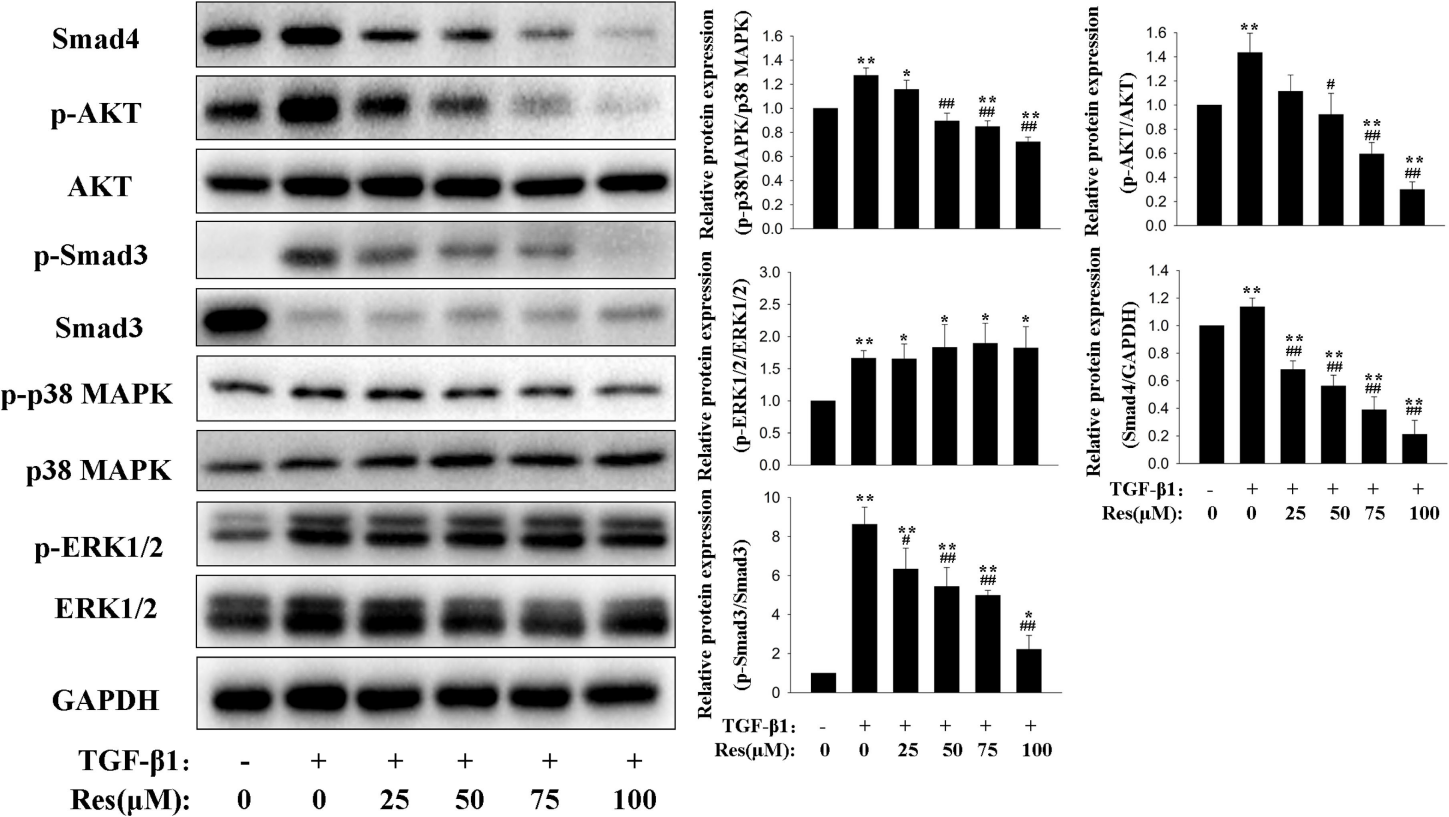
Effects of resveratrol on signaling pathways induced by TGF-β1 in HPFs. HPFs were treated with vehicle, TGF-β1 (10 ng/mL) only or plus different concentration of resveratrol for 48 hours. Representative Western blot images of Smad4, p-AKT, AKT, p-Smad3, Smad3, p-ERK1/2, ERK1/2, p-38 MAPK and p-p38 MAPK protein expression. Quantitative analysis of immunoblots with summary data from three independent experiments. Total protein levels were normalized to those of GAPDH (loading control). The levels of the phosphorylated proteins were normalized to their respective total protein levels. Error bars represent SEM. *P < 0.05, **P < 0.01, versus control; ^#^P < 0.05, ^##^P < 0.01 versus with TGF-β1, n = 3. Abbreviations: GAPDH, glyceraldehyde-3-phosphate dehydrogenase; HPF, human pterygium fibroblast; SEM, standard error of the mean; Res, resveratrol; TGF-β, transforming growth factor-β.

HPFs were treated indicated for 48 hours and the effects of resveratrol on the signaling pathways of TGF-β1-induced were detected by Western blotting. As the Fig. 2 shown, the expression protein level of p-AKT, p-p38 MAPK, p-ERK1/2 and p-Smad3 were dramatic increased after treatment of TGF-β1. However, the increased expression of p-AKT, p-Smad3, and p-p38-MAPK induced by TGF-β1 were inhibited by resveratrol in a dose-dependent manner in HPFs. The expression of p-ERK1/2 was not changed by Resveratrol in HPFs.

### TGF-β1 induces the fibrosis effects partly via PI3K/AKT, Smad3 and p38 MAPK signaling in HPFs

**Fig. 3:**
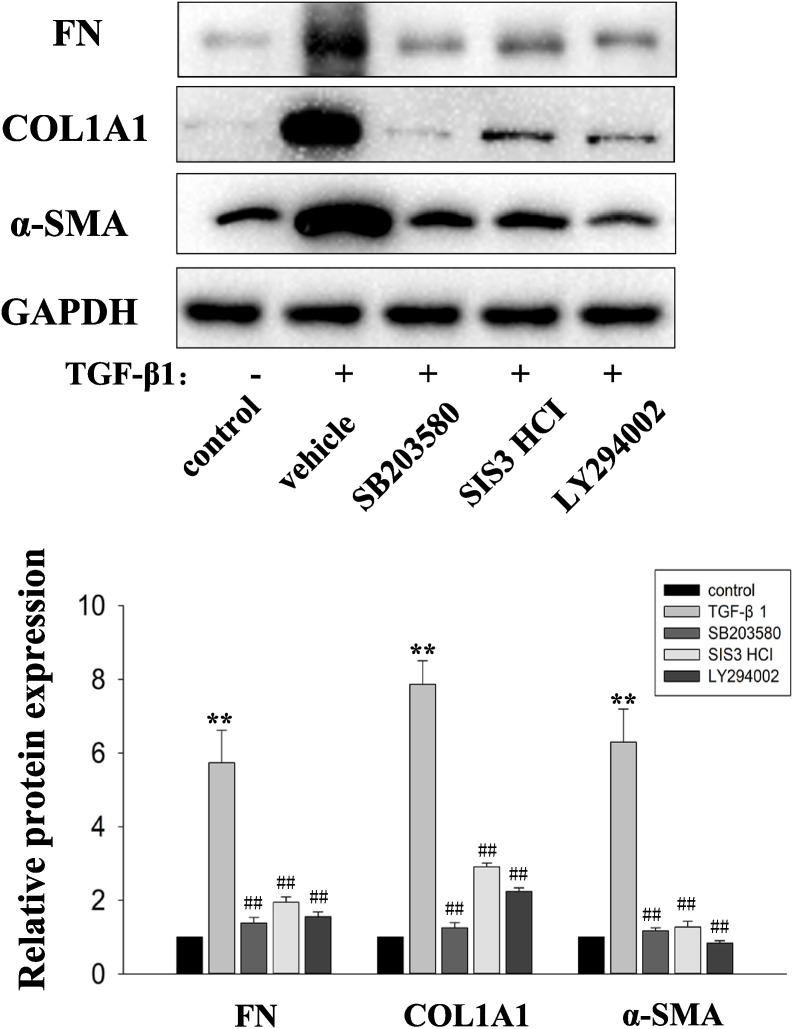
Effects of Smad3, PI3K/AKT and p38 MAPK inhibitors on the TGF-β1–induced HPFs. HPFs were treated with vehicle, TGF-β1 (10 ng/mL) only or plus PI3K/AKT inhibitor (LY294002, 30 µM) and p38 MAPK inhibitors (SB203580, 20 µM) and Smad3 inhibitor (SIS3 HCI, 20 µM) for 48 hours. Representative Western blot images of α-SMA, FN and COL1 protein expression. Quantitative analysis of immunoblots with summary data from three independent experiments. Total protein levels were normalized to those of GAPDH (loading control). Error bars represent SEM. *P < 0.05, **P < 0.01, versus control; ^#^P < 0.05, ^##^P < 0.01 versus with TGF-β1, n = 3. Abbreviations: α-SMA, α-smooth muscle actin; COL1, type 1 collagen; FN, fibronectin; GAPDH, glyceraldehyde-3-phosphate dehydrogenase; HPF, human pterygium fibroblast; SEM, standard error of the mean; Res, resveratrol; TGF-β1, transforming growth factor-β1.

To test whether AKT, Smad3 and p38 MAPK signaling pathways were involved in the TGF-β1-dependent fibrosis effects. HPFs were incubated with the indicated inhibitors with TGF-β1 (10 ng/mL) for 48 hours and the expression of relative proteins were tested by Western blot to explore the effects of inhibitors. The p38 MAPK signaling, TGF-β/Smad3 signaling and PI3K/AKT signaling were blocked with the inhibitor SB203580 (20 µM), SIS3 HCI (20 µM), and LY294002 (30 µM), respectively. As shown in Fig. 3, the expression levels of α-SMA, FN and COL1 were decreased by treatment with SB203580, LY294002 and SIS3 HCI compared with the TGF-β1 group. Collectively, this indicated that PI3K/AKT, Smad3 and p38 MAPK Signaling all involved the ECM synthesis and myofibroblast activation induced by TGF-β1 in HPFs.

### Resveratrol reduced the proliferation of HPFs by inducing cell apoptosis

**Fig. 4:**
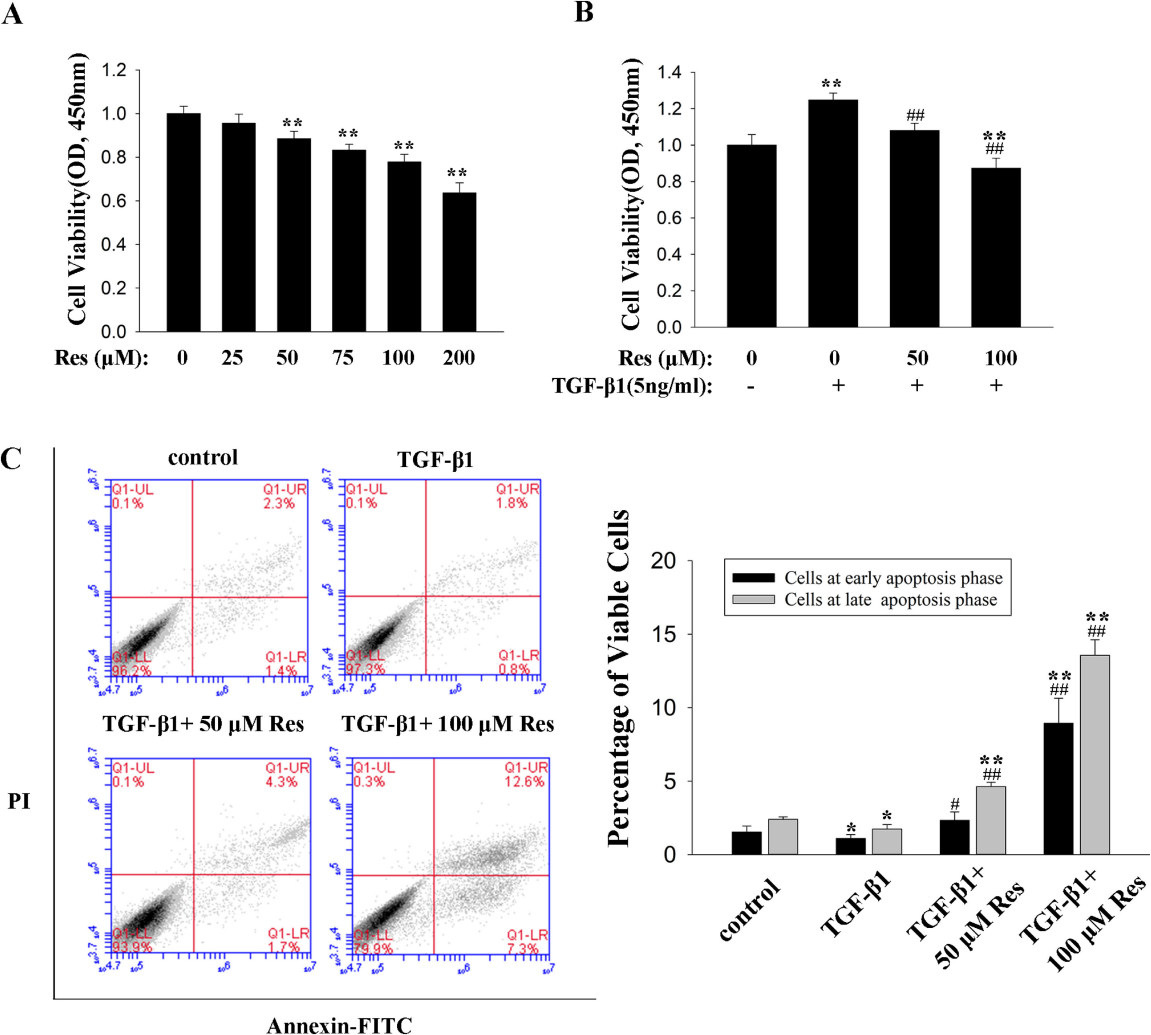
Effects of resveratrol on cell viability and apoptosis in HPFs. HPFs were treated with vehicle, TGF-β1 (10 ng/mL) only or plus different concentration of resveratrol for 48 hours. Quantitative representation of CCK-8 assay results after treatment with different concentrations of resveratrol only (A) or with TGF-β1 for 48 hours (B). (C) FACS results of different groups of HPFs treated as indicated for 48 hours. Quantitative analysis of the percentage of cell apoptosis with summary data from three independent experiments. Error bars represent SEM. *P < 0.05, **P < 0.01, versus control; ^#^P < 0.05, ^##^P < 0.01 versus with TGF-β1, n = 3. Abbreviations: CCK-8, Cell Counting Kit-8; FACS, fluorescence-activated cell sorting; FITC, fluorescein isothiocyanate; HPF, human pterygium fibroblast; PI, propidium iodide; Res, resveratrol; SEM, standard error of the mean; TGF-β1, transforming growth factor-β1.

HPFs were incubated with various concentration of resveratrol (0, 25, 50, 75, 100, 200 µM) or TGF-β (10 ng/mL) only or plus resveratrol (50, 100 µM) for 48 hours and the cells viability was detected by CCK-8. The CCK-8 assays results showed that TGF-β1 increased the proliferation ability of HPFs (Fig. 4B) and resveratrol treatment could significantly reduce the cell number dose dependently beginning with a concentration of 50 µM in the presence or absence of TGF-β1 (Fig. 4A, 4B). Then, the cellular apoptosis of HPFs were assessed by Annexin-V/PI staining. The results of fluorescence-activated cell sorting analysis showed that treatment with TGF-β1 decreased the cells at early apoptosis and late apoptosis, while treatment with resveratrol reversed the effects of TGF-β1 causing an increase percentage of distribution of early apoptosis and late apoptosis (Fig. 4C). Collectively, resveratrol may suppress the cellular proliferation partly by inducing cell apoptosis.

### Resveratrol suppressed cellular migration and contractile phenotype of HPFs induced by TGF-β1

**Fig. 5:**
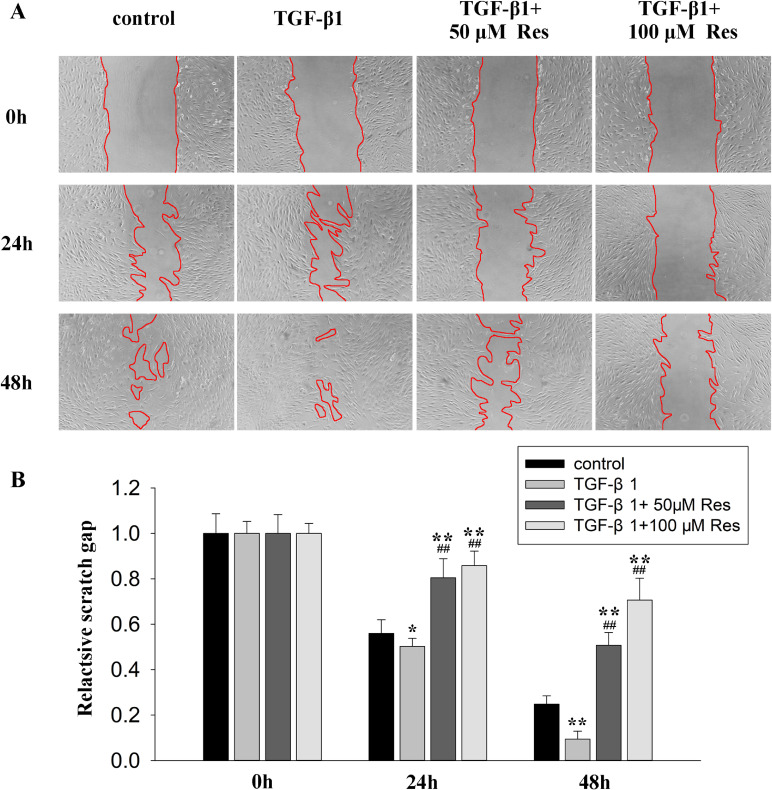
Effects of resveratrol on cell migration in HPFs. After wounding, HPFs were treated with vehicle, TGF-β1 (10 ng/mL) only or plus different concentration of resveratrol and wound closure was monitored by microscopy and photographed at the indicated times points. **(A)** Representative micrograph for each condition. Red lines define the boundary of migrating cells. **(B)** Quantitative analysis of the wound gap area with summary data from three independent experiments. Error bars represent SEM. *P < 0.05, **P < 0.01, versus control; ^#^P < 0.05, ^##^P < 0.01 versus with TGF-β1, n = 3. Abbreviations: HPF, human pterygium fibroblast; SEM, standard error of the mean; Res, resveratrol; TGF-β1, transforming growth factor-β1.

The effects of resveratrol on cell migration in TGF-β1-treated HPFs were studied by wound healing assay. As the wound healing assay result shown, TGF-β1 enhanced the wound gaps closure compared to the control group which indicted the promotion effects of TGF-β1 in cell migration. However, treatment with resveratrol markedly suppressed the closure of wound gaps (Fig. 5). Those results suggest that resveratrol inhibits TGF-β1-induced wound closure by suppressing cell migration in HPFs.

**Fig. 6:**
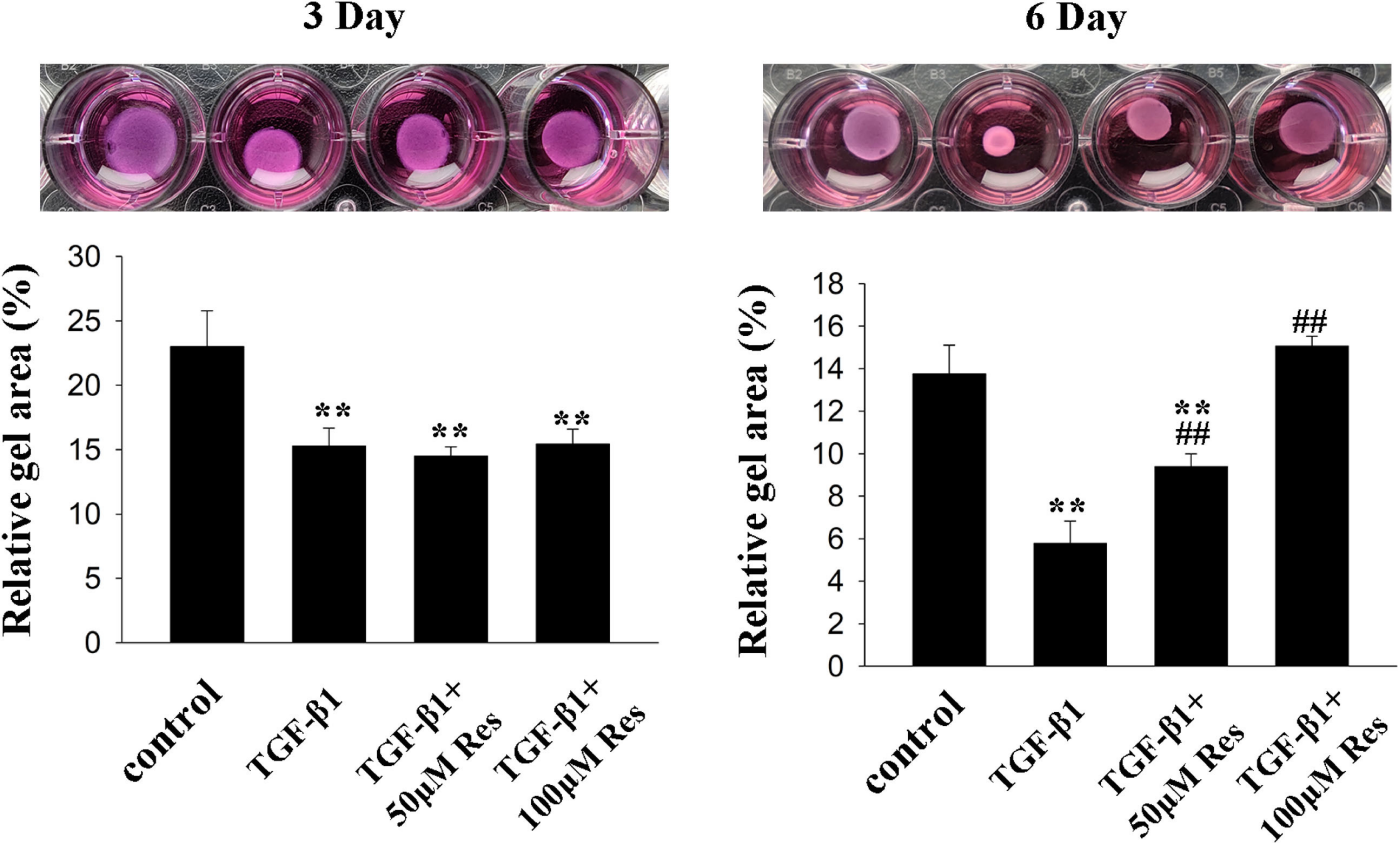
Effects of resveratrol on the contractile phenotype of HPFs. HPF-embedded collagen gels were prepared and placed in medium with vehicle, TGF-β1 (10 ng/mL) only or plus different concentration of resveratrol. Images were captured at the indicated time points. Quantitative analysis of collagen area compared to the surface area of well with summary data from three independent experiments. Error bars represent SEM. *P < 0.05, **P < 0.01, versus control; ^#^P < 0.05, ^##^P < 0.01 versus with TGF-β1, n = 3. Abbreviations: HPF, human pterygium fibroblast; SEM, standard error of the mean; Res, resveratrol; TGF-β1, transforming growth factor-β1.

To further confirm the anti-fibrotic effects of resveratrol, the collagen gel contraction assays in TGF-β1 treated HPFs were conducted to assess the contractile phenotype of HPFs. As the results showed, treatment of HPFs-embedded collagen with TGF-β1 significantly reduced the collagen gel area at 3 days. While treatment with 50 µM resveratrol markedly attenuated the contraction induced by TGF-β1; 100 µM resveratrol not only prevented the effect stimulated by TGF-β1, but also slightly reversed the basal contraction effects (Fig. 6). Altogether, resveratrol could suppress contractile phenotype of HPFs.

## Discussion

The pathology of pterygium involves excessive ECM deposition, cell hyperproliferation, overexpression of anti-apoptotic factors, and chronic inflammation. Surgical excision is the standard therapy for pterygium, but recurrence remains a concern, leading to increased surgical difficulty and postoperative complications [19]. Resveratrol has gained attention for treating various ocular diseases [20], such as age-related macular degeneration [21], cataract [22], diabetic retinopathy [23]. However, its effect on ocular fibrotic disorders like glaucoma, corneal scarring, and pterygium is still unknown. In this study, we found resveratrol inhibited the expression of α-SMA, FN, and COL1, and suppressed cellular migration, proliferation, and the contractile phenotype induced by TGF-β1 in HPFs.

Cellular hyperproliferation is a significant feature in pterygium [24]. and its recurrence is associated with excessive wound healing after surgery. Anti-proliferative drugs like mitomycin C have been used to inhibit pterygium recurrence in clinical applications and cell models [25–27]. Anti-apoptotic mechanisms also play a role in the etiopathogenesis of pterygium [28]. Our cell viability assays showed that resveratrol inhibited cellular proliferation by inducing apoptosis in HPFs. Similar to studies in keloid fibroblasts, resveratrol inhibited cellular proliferation and promoted apoptosis by targeting HIF-α [29]. However, the role of resveratrol can vary depending on the conditions and cell types. Resveratrol has been reported to attenuate apoptosis in certain cell types while attenuating oxidative stress in others [30]. Considering its antioxidant effects, resveratrol may benefit normal conjunctival tissues exposed to UV radiation.

Fibrosis involves complex intercellular networks [31] with TGF-β-triggered cellular transduction playing a vital role in fibrotic pathogenesis [32]. Our study demonstrated that blocking the PI3K/AKT, p38 MAPK, or TGF-β/Smad3 pathways suppressed ECM protein synthesis and myofibroblast activation induced by TGF-β1. TGF-β1 is overexpressed in pterygium tissues [7], and involved in postoperative wound healing. Targeting TGF-β-mediated Smad signaling has shown promise in many fibrotic diseases [33]. The canonical Smad2/3 signaling pathway has also been implicated in ECM deposition and myofibroblast activation in pterygium [27, 34]. Additionally, the PI3K/AKT and p38 MAPK pathways have been confirmed to participate in the fibrogenesis of HPFs [35, 36].

Our study further showed that resveratrol inhibited the phosphorylation of AKT, p38 MAPK, and Smad3 in HPFs. The anti-fibrotic property of resveratrol via Smad3 signaling has also been validated by other studies [37–39]. However, the exact mechanisms through which resveratrol regulates these signaling pathways are still unclear. Resveratrol has been reported to ameliorate renal fibrosis by enhancing the binding between Sirt1 and Smad3, reducing acetylation levels of Smad3 [38]. Activation of Sirt3 by resveratrol has also been shown to inhibit TGF-β/Smad3 signaling in cardiac fibroblasts [39]. However, the activation of Sirt1 may play positive roles in renal fibrogenesis [40]. Therefore, the role of Sirt1 activation may vary in fibroblasts and needs further evidence. Apart from the TGF-β/Smad3 pathway, our study demonstrated that resveratrol also inhibits the non-canonical pathways PI3K/AKT and p38 MAPK, as reported in several studies [14, 41, 42].

To mimic the clinical presentation of astigmatism caused by pterygium, we utilized a collagen contraction assay to study ECM remodeling and wound contraction during wound healing [43]. Resveratrol has previously been shown to inhibit collagen gel contraction in various cell types [44]. Consistent with this, our study demonstrated that resveratrol fully blocked the TGF-β1-induced contractile phenotype of HPFs.

Our study revealed that the antifibrotic effect of resveratrol in HPFs were in part contributed to regulate the PI3K/AKT, p38 MAPK and Smad3 signaling. Nevertheless, there are certain limitations to this study. Further experiments are necessary to fully understand the mechanisms underlying the antifibrotic effects of resveratrol. Additionally, the effects of resveratrol on cell proliferation and apoptosis in normal conjunctival fibroblasts need to be confirmed to assess toxicity and safety for clinical research. Furthermore, the effects of resveratrol on pterygium epithelial cells and normal conjunctival epithelial cells should also be investigated.

In conclusion, our study demonstrated that resveratrol effectively inhibits TGF-β1-induced synthesis of ECM proteins, myofibroblast activation, cell migration, contractile phenotype, and cellular proliferation by inducing apoptosis in HPFs. These findings suggest that resveratrol may be a potential adjuvant drug to prevent pterygium recurrence after surgery.

## Abbreviations

HPFs: human pterygium fibroblasts
TGF-β1: transforming growth factor-beta1
COL1: type 1 collagen
α-SMA: α-smooth muscle actin
UV: ultraviolet
ECM: extracellular matrix
Res: Resveratrol
FBS: fetal bovine serum
DMSO: dimethyl sulfoxide
BCA: bicinchoninic acid
SDS-PAGE: sodium dodecyl sulfate polyacrylamide gel electrophoresis
PVDF: polyvinylidene fluoride membrane
TBST: Tris-buffered saline with 0.1% Tween-20
ECL: enhanced chemiluminescence
OD: optical density
FITC: fluorescein isothiocyanate
PI3K: phosphatidylinositol-3-kinase
AKT: protein kinase B
p38 MAPK: mitogen-activated protein kinase
Sirt1: silent information regulator sirtuin 1

## Declaration

### Availability of data and materials

No additional data are available.
